# Supplementary material for: Decoding biomaterial-associated molecular patterns (BAMPs): influential players in bone graft-related foreign body reactions
Source: PeerJ. 2025 Apr 22;13:e19299. doi: 10.7717/peerj.19299 (PMC12024449; doi:10.7717/peerj.19299)

Fig. S1. Classification of bone grafting biomaterial. The various types of bone grafting biomaterials are categorized based on their source of origin. (Flowchart created using Biorender.com)

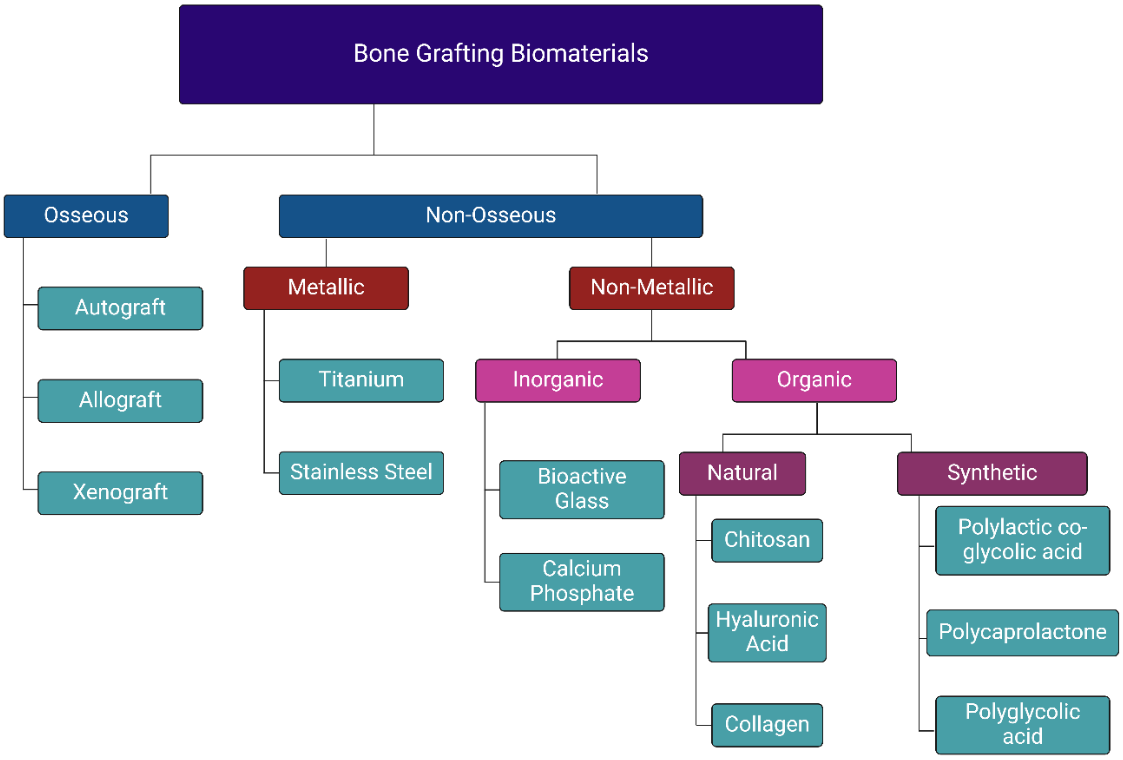

Supplement: Supplemental Information 1 — The various types of bone grafting biomaterials are categorized based on their source of origin. Flowchart created using Biorender.com. [file peerj-13-19299-s001.pdf]
