# Supplementary material for: Decoding biomaterial-associated molecular patterns (BAMPs): influential players in bone graft-related foreign body reactions
Source: PeerJ. 2025 Apr 22;13:e19299. doi: 10.7717/peerj.19299 (PMC12024449; doi:10.7717/peerj.19299)
Supplement: Supplemental Information 2 [file peerj-13-19299-s002.docx]

**Table S1.** Outlines the advantages and disadvantages of various bone grafting biomaterials.

| Bone Grafting Material | Advantages | Disadvantages | References |
| --- | --- | --- | --- |
| Autograft | - Regarded as the gold standard.  - No risk of immune rejection.  - Excellent integration potential.  - Demonstrates osteogenic, osteoinductive, and osteoconductive qualities (includes live cells and growth factors). | - Unsuitable for larger defects because of the limited availability of the patient’s own bone.  - Harvesting from a donor site can lead to complications such as pain, infection, scarring, and decreased functionality at the extraction location. | (Oryan et al., 2014; Titsinides et al., 2019) |
| Allograft | - Avoids donor-site morbidity.  - Primarily osteoconductive, it may also retain osteoinductive properties.  - A source of type I collagen and bone morphogenic proteins (BMPs).  -Decreased operative time and blood loss.  - Available in cancellous, cortical, osteochondral, and demineralized bone matrix. | - Infection risks are associated with allografts.  - They have no osteogenic potential.  - Sterilization and storage processes can affect their osteoconductive and osteoinductive properties. | (Sohn & Oh, 2019; Titsinides et al., 2019) |
| Xenograft | - Deproteinized xenografts have outstanding physicochemical properties.  - Can be produced in large quantities with relatively low processing costs.  - Hydroxyapatite (HA) is the main inorganic composition, which improves osteoconductive properties. | - Insufficient organic materials, resulting in reduced osteoinductive properties.  - Potential risk of disease transmission.  - Risk associated with graft rejection. | (Amid et al., 2020; Titsinides et al., 2019) |
| Titanium (Metallic) | - Biometals like stainless steel and titanium alloys have outstanding mechanical strength and impressive plastic toughness.  - Promotes osseointegration.  - Feature high resistance to corrosion. | - Titanium particles and ions can accumulate in peri-implant tissues, leading to inflammatory responses and bone resorption.  - Instances of inadequate osseointegration documented. | (Pałka & Pokrowiecki, 2018; Webber et al., 2021) |
| Hydroxyapatite (HA)  Tricalcium phosphate (TCP) | - HA has a porous structure that improves bio-absorbability and promotes effective bone growth.  - Due to its chemical resemblance to natural bone, it offers outstanding bioactivity, biocompatibility, and osteoconductivity.  - HA is resistant to sterilization processes, guaranteeing consistent quality, and is cost-effective.  - Combination of graphene oxide (GO) and HA biopolymer bone scaffolds has shown improved mechanical properties, bioactivity, and osteoconductivity.  - Does not produce a metabolite that hinders osteogenesis and significantly has decreased FBR potential.  - Highly osteoconductive and closely resembles the chemical composition of human bone.  - β-TCP shows good biodegradability along with its bioactive properties. | - HA remains in the body for an extended period, potentially hindering bone remodeling and prolonging mechanical weakness in the newly formed bone.  - Exhibit high brittleness and low strength.  - Low elasticity and poor fracture toughness.  - Excessively high stiffness, reducing reliability and resistance to damage.  - Not indicated in larger defects due to the fragility of calcium phosphate. | (Feng et al., 2023; Pina et al., 2018; Shuai, Peng, et al., 2021; Shuai, Yang, et al., 2021; Sohn & Oh, 2019) |
| Chitosan | - Exhibit positive charge, biocompatibility, osteoconductivity, biodegradability, and low toxicity.  -Ideal 3D scaffold for bone tissue engineering.  - The positive charge improves cell attachment and facilitates cellular uptake, thus enhancing the efficiency of bone regeneration processes. | - Limited mechanical properties, rapid degradation rates, and low osteoinductivity hinder their use in bone tissue engineering.  - Insufficient mechanical strength poses a significant challenge for employing pure chitosan scaffolds in load-bearing situations applications. | (Aibani et al., 2021; Signorini et al., 2023) |
| Hyaluronic Acid | - Supports bone growth by serving as a carrier for osteoinductive compounds.  -It promotes uniform distribution, increases bone density, improves scaffold morphology, and enhances mineralization. | -These graft materials often require an extended period for complete healing.  -They are associated with a limited percentage of new bone regeneration. | (De Risi et al., 2015; Lorenzi et al., 2024) |
| Collagen | - Similarity to extracellular matrix elements ensures high cytocompatibility and reduced antigenicity, leading to a slight immune response.  - Enhances cell attachment, growth, and differentiation, supporting tissue regeneration. | - Collagen exhibits low stability after implantation, potentially affecting its long-term functionality performance. | (Kaczmarek-Szczepańska et al., 2023) |
| Poly-l-lactic acid | - Enhances cell adhesion, growth, and differentiation, making bone regeneration effective.  - The combination of biocompatibility and biodegradability renders PLLA a highly suitable material for tissue engineering.  - The addition of PGA to the HAP/PLLA scaffold can accelerate the degradation of PLLA via hydrolysis chains. | - 6 months and 3 years to fully degrade, making it suitable for long-term applications.  - Slow degradation occurs due to the hydrophobic methyl group in its polymer backbone. | (Feng et al., 2018; Ge et al., 2018; Shuai et al., 2021) |
| Polycaprolactone | - Biocompatible, non-cytotoxic, low-cost, biodegradable polymer.  - Superior mechanical properties and robust plasticity.  - High molecular weight enhances mechanical strength, making it suitable for bone tissue engineering applications. | - Hydrophobic nature of PCL limits cell attachment, affinity, and proliferation.  - Combining PCL with other polymers or ceramics or modifying its surface can enhance its properties for specific applications. | (Dwivedi et al., 2020; Liang et al., 2024) |
| Polyglycolic acid | - Degrades within a few weeks due to its high hydrophilicity, making it ideal for short-term applications.  - Good biosafety, biodegradability, and formability make it suitable for bone regeneration.  - Provides straightforward quality control and features low production costs, making it highly efficient for various purposes and applications. | - Insufficient strength and acidic degradation products can cause aseptic inflammation reactions. | (Feng et al., 2023; Kalitheertha Thevar et al., 2019) |
| Polyetheretherketone (PEEK) | - Non-mutagenic, non-cytotoxic, and does not cause adverse reactions or release harmful substances in human tissues.  - Low elastic modulus of 3–4 GPa, which is close to that of human cortical bone at 7–30 GPa.  - Minimizes stress shielding in bone-related context application.  - Elastic modulus and mechanical strength close to human cortical bone.  - PLGA blending with PEEK enhances its biodegradability. | - PEEK's biological inertness and weak antibiological properties lead to unsatisfactory results in certain applications.  - The lack of osteointegration and antibacterial properties may cause aseptic loosening, infectious complications, early implant revisions, and potential implant failure.  - Do not degrade; hence, possess low bioactivity and biodegradability. | (Feng et al., 2018; Wickramasinghe et al., 2022) |
